# Expression and role of FKBPL in lung adenocarcinoma

**DOI:** 10.7150/jca.87758

**Published:** 2024-01-01

**Authors:** Lili She, Xingsong Zhang, Rong Shen, Song He, Xiaobing Miao

**Affiliations:** 1 Department of Pathology, Affiliated Tumour Hospital of Nantong University, Nantong, China.; 2 Department of Pathology, Nantong Sixth People's Hospital, Nantong, China.

**Keywords:** lung adenocarcinoma, FKBPL, overall survival, cell proliferation, cell apoptosis

## Abstract

Dysregulated expression of FK506-binding protein like (FKBPL) has been demonstrated to play crucial roles in tumour development. However, the role of FKBPL in lung adenocarcinoma (ADC) remains unclear. Using immunohistochemical staining, we showed that FKBPL expression was significantly lower in lung ADC than the normal tissues (*P* < 0.0001). Patients with well or moderately differentiated tumours have higher FKBPL expression compared with patients with poor differentiated tumours (*P* = 0.037). However, no significant associations were found between FKBPL expression and other clinicopathological variables (*P* > 0.05 for all). Cox univariate analysis showed that high FKBPL expression was correlated with prolonged overall survival (OS) (*P* = 0.010). Kaplan-Meier analysis further confirmed that the FKBPL-low group showed a significantly shorter OS than the FKBPL-high group (*P* = 0.0081). FKBPL expression was not shown as an independent prognostic factor for OS in the multivariate analysis (*P* = 0.063). Moreover, our study demonstrated that FKBPL could suppress the proliferation of lung ADC cells by delaying cell cycle G1/S phase transition. In addition, FKBPL resulted in increased apoptosis in lung ADC cells. Using the Human Apoptosis Array Kit, we observed that overexpression of FKBPL in lung ADC A549 cells significantly decreased the anti-apoptotic proteins, including heat shock protein 32 (HSP32), heat shock protein 27 (HSP27), and paraoxonase-2 (PON2). FKBPL depletion significantly attenuated the pro-apoptotic protein, phospho-p53 (S46), in lung ADC H1975 cells. These new findings provide an experimental basis for further theoretical investigation of lung ADC.

## 1. Introduction

Lung cancer is the leading cause of cancer mortality in China, and adenocarcinoma (ADC) is the most common histological subtype 1, 2. Lung ADC is heterogeneous in its clinical presentation, morphology, molecular features, biological behaviour, and treatment options 3. Treatment options for lung ADC include surgery, radiation therapy, chemotherapy, targeted therapy and immunotherapy 4. Tyrosine kinase inhibitors (TKIs) have improved the outcome of patients harboring epidermal growth factor receptor (*EGFR*)-activating mutations. More molecular targets such as anaplastic lymphoma kinase (*ALK*), ROS proto‐oncogene 1, receptor tyrosine kinase (*ROS1*), and v-raf murine sarcoma viral oncogene homolog B1 (*BRAF*) have shown promising anti-tumor efficacy. However, the onset of primary and acquired resistances continue to be the main obstacle to further improve clinical outcomes 5. Therefore, identifying novel biomarkers and molecular targets is integral for improving patient outcomes.

FK506-binding protein like (FKBPL) belongs to the immunophilin protein family, a group of conserved proteins binding to immunosuppressive drugs, such as FK506, rapamycin, and cyclosporin A 6, 7. Immunophilins are involved in many cellular processes, such as cell signalling, cell cycle progression, metabolic activity and apoptosis 8. FKBPL has been shown to play a critical role in regulating estrogen receptor (ER), androgen receptor (AR), glucocorticoid receptor (GR) and inflammatory signalling, cancer stem cell differentiation, and inhibition of angiogenesis 9, 10. Recently, FKBPL is described as a dual leucine zipper kinase (DLK)-interacting protein that regulates DLK degradation via the lysosome and neuronal responses to axon injury 7. Lin et al. 11 reported that miR-183-5p promotes the proliferation and migration of vascular smooth muscle cells by reducing FKBPL expression. Substantial evidence indicates that FKBPL functions as a tumour suppressor. FKBPL knockdown in breast cancer cell lines leads to an increase in Nanog/Oct4/Sox2 and an increase in cancer stem cell number 12. Ectopic expression of FKBPL inhibits the proliferation of breast cancer cells by stabilizing the cyclin-dependent kinase (CDK) inhibitor, p21. In addition, FKBPL overexpression renders breast cancer cells more sensitive to tamoxifen. Furthermore, breast cancer patients with high FKBPL expression are more likely to have increased overall survival (OS) and distant metastasis-free survival 13, 14. FKBPL expression is significantly decreased in endometrioid endometrial carcinoma in comparison to benign endometrial hyperplasia tissue, with a high predictive value of malignancy. FKBPL expression is positively correlated with ER expression and negatively correlated with VEGF-A expression 15. A recent study on high-grade serous ovarian cancer shows that patients with high endogenous tumour expression of FKBPL have an increased progression-free survival 16. However, the prognostic role of FKBPL in patients with lung ADC, and the possible function of FKBPL in the development and progression of lung ADC remains unclear. The aim of this study was to investigate the biological function and clinical significance of FKBPL in lung ADC. Our findings suggest that FKBPL can suppress cell proliferation by delaying cell cycle G1/S phase transition, and promote the apoptosis of lung ADC cells.

## 2. Materials and methods

### 2.1 Patients and tissue samples

Two hundred and twenty-two cases of lung ADC, diagnosed between January 2014, and December 2018 at the Affiliated Tumour Hospital of Nantong University, were included in this study. The inclusion criteria were as follows: (1) patients were histopathologically confirmed as lung ADC; (2) treatment naïve; (3) with complete follow-up, clinical and pathological information. The exclusion criteria were as follows: (1) with other serious diseases; (2) received other treatments, including radiation therapy, chemotherapy, molecularly targeted therapy, or immunotherapy before specimen collection. H&E slides of all cases were reviewed according to the 2021 World Health Organization (WHO) diagnostic criteria. This study was approved by the Ethics Committee of the Affiliated Tumour Hospital of Nantong University.

### 2.2 Immunohistochemistry and evaluation of immunohistochemistry

Tissue microarray (TMA) was constructed as described previously 17. The TMA was constructed using a manual tissue puncher/arrayer. After reviewing the H&E slides of all cases, the representative slides and formalin-fixed paraffin blocks from each case were selected. For each sample, a 3.0 mm diameter core of tissue was rearranged into recipient blocks. Immunohistochemistry was performed according to standard protocols. Briefly, the 4-μm-thick sections were deparaffinized with xylene and dehydrated using gradient alcohol. Antigen retrieval, elimination of the endogenous peroxidase activity, and blocking were carried out according to standard protocols. The sections were then incubated with FKBPL antibody (1:100 dilution, Proteintech Group, Wuhan, China) at 4 °C overnight. Following washing, slides were incubated with HRP-conjugated secondary antibody (Dako REAL Envision Detection System, Dako, Glostrup, Denmark) for 30 min and subsequently with DAB chromogen (Dako REAL Envision Detection System), and counterstained with hematoxylin. Assessment of immunohistochemical staining was performed semi-quantitatively using the Histo-score (H-score). H-scores were calculated to give a score from 0 to 300 based on staining intensity and proportions of stained cells as previously described 18. Briefly, staining intensity was evaluated as negative (0), weak (1+), moderate (2+), and strong (3+). The percent of cells within each tissue core stained at each intensity was recorded. The H-score was determined as: H-score = 0× (% at 0) + 1× (% at 1+) + 2× (% at 2+) + 3× (% at 3+). The patients were dichotomized into low (H-score <80) and high (H-score ≥80) FKBPL expression level groups by the optimal cut-off threshold calculated by X-tile software (Yale University).

### 2.3 Cell culture, and transient transfection

The H1975 human lung ADC cell line was kindly provided by Stem Cell Bank, Chinese Academy of Sciences. H1975 cells were routinely maintained in RPMI 1640 medium (GIBCO, Grand Island, NY, USA) supplemented with 10% fetal bovine serum (FBS, GIBCO). The A549 human lung ADC cell line was purchased from Cobioer Biosciences Company (Nanjing, China). A549 cells were incubated in F-12K medium (GIBCO) supplemented with 10% FBS.

The FKBPL overexpression (OE) plasmid and FKBPL small hairpin RNA (shRNA) expression plasmids were synthesized by GeneChem Co., Ltd. (Shanghai, China). FKBPL targeting sequences were: CAGAACTATTTCGAGCTGGGA (shFKBPL#1), GCTTTGTCAAGAAGATCGTAA (shFKBPL#2). A non-specific shRNA with a sequence of TTCTCCGAACGTGTCACGT served as the negative control. Transient transfection of plasmids was performed using Lipofectamine 2000 (Invitrogen, Shanghai, China) following the manufacturer's protocol.

### 2.4 Antibodies, and western blot

Equal amounts of total protein were loaded and run on SDS-polyacrylamide gel electrophoresis (SDS-PAGE) and transferred to polyvinylidene fluoride (PVDF) membranes (Roche, Basel, Switzerland). The membranes were blocked with 5% skim milk (in TBS-T) for 2 hours at room temperature and then probed with the primary antibodies overnight at 4°C. The next day the membranes were washed three times with TBS-T for 5 min each and incubated with the relevant secondary antibodies for an hour at room temperature. After washed three times with TBS-T, the membranes were visualized using enhanced chemiluminescence (ECL) reagents (NCM Biotech, Suzhou, China) and ChemiDoc Touch Imaging System (Bio-rad, Hercules, CA, USA). Antibodies and dilutions were as follows: anti-FKBPL antibody (1:2000 dilution, Proteintech Group), anti-Flag-M2 antibody (1:1000 dilution, Sigma-Aldrich, St Louis, MO, USA), anti-Cyclin E1 antibody (1:1000 dilution, Proteintech Group), anti-GAPDH antibody (1:5000 dilution, Proteintech Group), Peroxidase AffiniPure Goat Anti-Rabbit IgG (H + L) (1:10000 dilution, Jackson ImmunoResearch Laboratories, West Grove, PA, USA), Peroxidase AffiniPure Goat Anti-Mouse IgG (H + L) (1:10000 dilution, Jackson ImmunoResearch Laboratories).

### 2.5 Colony formation assay

Cells were trypsinized, counted, and seeded for colony formation assay in 35-mm culture dishes at a density of 1×10^3^ cells/dish. During colony growth, the culture medium was replaced every 3 days for 15 days. Colonies were fixed in 4% paraformaldehyde (Beyotime Biotech, Shanghai, China), and stained with 0.1% crystal violet (Beyotime Biotech).

### 2.6 Cell cycle analysis

For the cell cycle analysis, cells were harvested by trypsinization, and fixed with 70% ethanol overnight at -20 °C. The fixed cells were then centrifuged to remove ethanol, washed with PBS and resuspended in 0.5 mL of PI/RNase staining buffer (BD Biosciences, San Jose, CA, USA) for 15 min at room temperature in the dark. Cell cycle distributions were determined on the BD FACSCanto II flow cytometer (BD Biosciences).

### 2.7 Apoptosis detection assay

For apoptosis analysis, cells were stained with APC Annexin V and 7-AAD (Biolegend, San Diego, CA, USA) according to the manufacturer's protocol. Briefly, cells were washed twice with cold BioLegend's cell staining buffer, and resuspend at 1.0 × 10^7^ cells/mL in Annexin V binding buffer. Cells were then placed into 5 mL test tubes, and stained with 5 µL of APC Annexin V and 5 µL of 7-AAD viability staining solution. After gently vortexing, the cells were incubated 15 min at room temperature in the dark. Thereafter, cells were incubated with 400 µL of Annexin V binding buffer and then subjected to flow cytometric analysis.

### 2.8 Apoptosis antibody array analysis

The expression profiles of apoptosis-related proteins were analyzed using the Proteome Profiler Human Apoptosis Array Kit (ARY009) according to the manufacturer's instructions (R&D Systems, Minneapolis, MN, USA). Briefly, the cells were lysated using the lysis buffer. Membranes containing immobilized antibodies were blocked with array buffer 1 for 1 h at room temperature and incubated with the cell lysates overnight at 4 °C. The membranes were then washed with wash buffer and incubated with detection antibody cocktail for 1 h and streptavidin-HRP for 30 min at room temperature. Protein expression was visualized using chemi reagents and the ChemiDoc Touch Imaging System.

### 2.9 Human protein atlas and Kaplan-Meier plotter database analysis

The Human Protein Atlas (https://www.proteinatlas.org/) is a website that involve immunohistochemistry-based expression data for distribution and expression of 20 tumour tissues 19. We used the Human Protein Atlas database to explore the prognostic value of FKBPL in lung ADC. We also used an online Kaplan-Meier plotter database (http://kmplot.com) to explore the value of FKBPL as a novel prognostic biomarker in lung ADC 20.

### 2.10 Statistical analysis

The associations between FKBPL expression and clinicopathological parameters were evaluated by Pearson's chi-square test. OS was estimated using the Kaplan-Meier method and compared with the log-rank test. Cox proportional hazards regression models were applied to evaluate the potential independent prognostic factors. Comparison between groups was evaluated by Student's t-test. *P* values of less than 0.05 were regarded as statistically significant. All statistical analysis was performed using SPSS 23.0 and GraphPad Prism 6.

## 3. Results

### 3.1 FKBPL expression in lung ADC tissues

**Figure 1:**
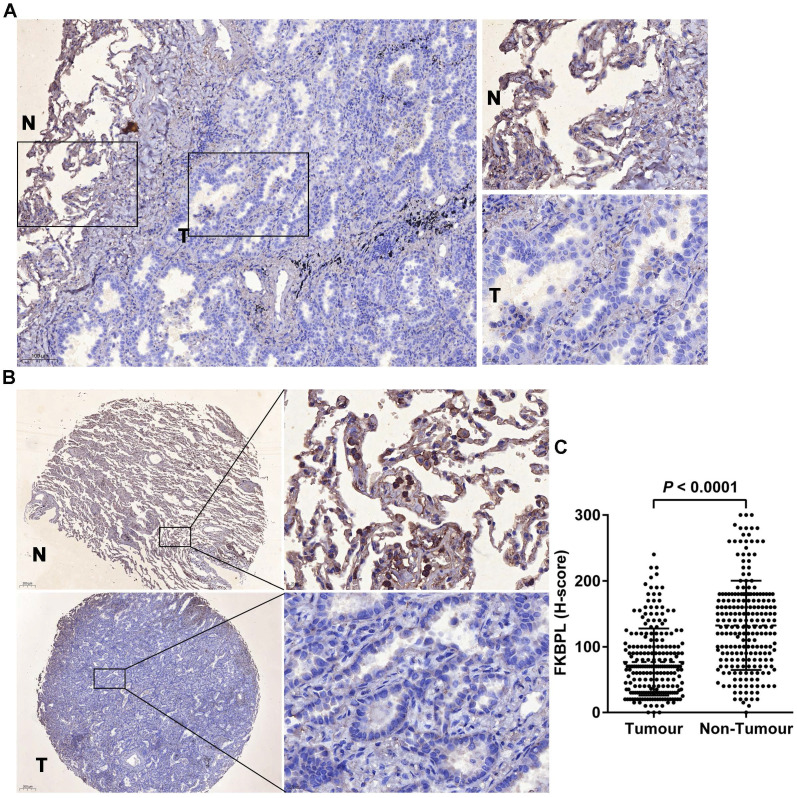
FKBPL was decreased in lung ADC compared with the matching normal tissues. (A) Representative image of immunohistochemical staining of FKBPL in whole section. 'T' represents tumour tissue and 'N' represents non-tumour lung tissue. (B) Representative images of immunohistochemical staining of FKBPL in TMAs. 'T' represents tumour tissue and 'N' represents non-tumour lung tissue. (C) Scatter plots showing a statistical analysis of FKBPL expression in tumour tissue and non-tumour lung tissue.

Immunohistochemical staining of the whole tissue sections showed that FKBPL was predominantly localized in the cytoplasm, and its expression levels were decreased in tumours compared with the matching normal tissues (Fig. 1A). The preliminary finding was confirmed in TMA which showed that FKBPL was significantly lower in lung ADC (H-score, 76.71±3.42) than the normal tissues (H-score, 132.40±4.54) (*P* < 0.0001; Fig. 1B and C). Of the 222 lung ADC patients, 92 (41.4%) cases had high FKBPL expression and 130 (58.6%) cases had low FKBPL expression. FKBPL expression was associated with tumour differentiation (*P* = 0.037). Patients with well or moderately differentiated tumours had higher FKBPL expression compared with patients with poor differentiated tumours. However, no significant associations were found between FKBPL expression and age, gender, smoking status, T stage, N stage, TNM stage, lymphovascular invasion, and STAS (*P* > 0.05 for all; Table 1).

### 3.2 Reduced FKBPL expression predicts poor outcomes

**Figure 2:**
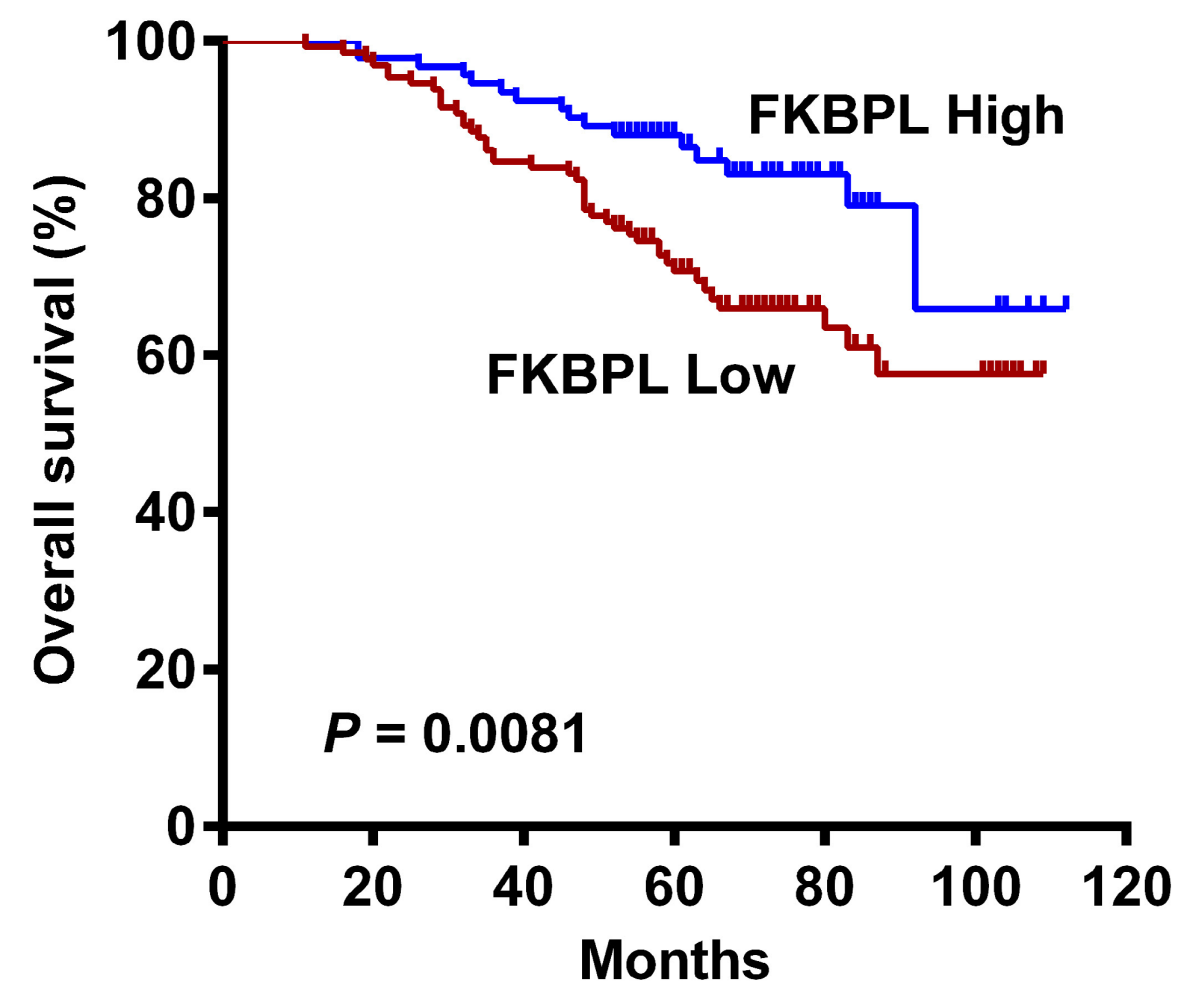
Kaplan-Meier curves for OS in lung ADC patients according to FKBPL expression status. The FKBPL-low group showed a significantly shorter OS than the FKBPL-high group (log-rank *P* = 0.0081).

No significant associations were found between FKBPL expression and OS in patients with lung ADC according to the Human Protein Atlas (Supplementary Fig. 1) and Kaplan-Meier plotter (Supplementary Fig. 2) databases. However, our univariate Cox analysis showed that high FKBPL expression was correlated with prolonged OS (HR = 0.471, 95% CI = 0.265 to 0.835, *P* = 0.010; Table 2). Kaplan-Meier analysis further confirmed that the FKBPL-low group showed a significantly shorter OS than the FKBPL-high group (*P* = 0.0081; Fig. 2). In univariate analysis, tumour differentiation (*P* < 0.001), T stage (*P* = 0.005), N stage (*P* < 0.001), lymphovascular invasion (*P* = 0.047), and STAS (*P* = 0.011) also showed a significant impact on OS. However, no significant associations were found between age, gender, smoking status and OS (*P* > 0.05 for all). Multivariate analysis demonstrated that T stage (*P* = 0.026) and N stage (*P* < 0.001) independently predicted OS. FKBPL expression was not an independent prognostic factor for OS (*P* = 0.063; Table 2).

### 3.3 FKBPL suppresses the proliferation of lung ADC cells

**Figure 3:**
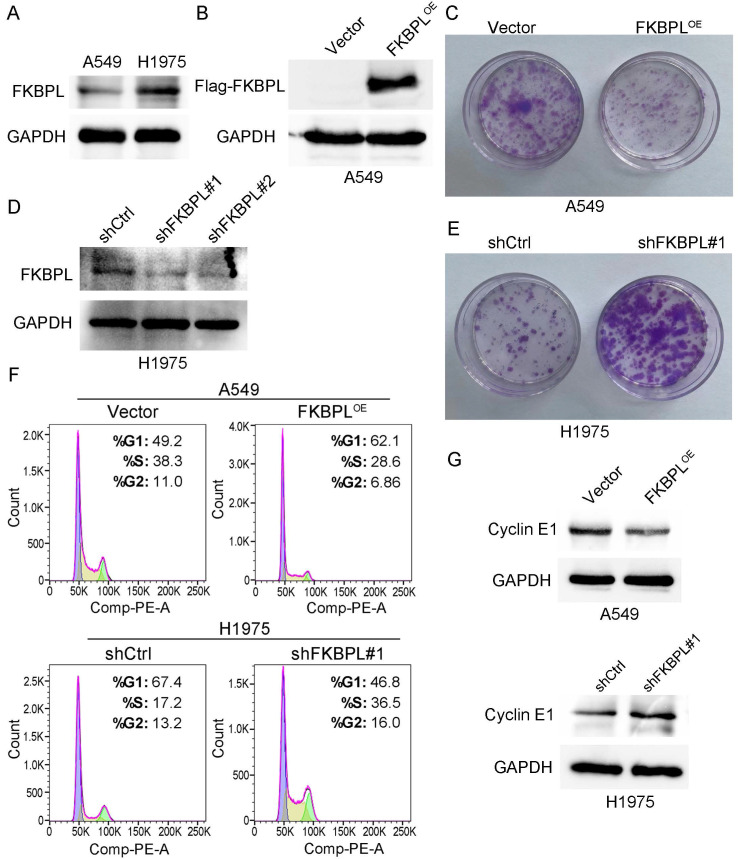
FKBPL suppresses the proliferation of lung ADC cells. (A) Protein levels of FKBPL in two lung ADC cell lines, A549 and H1975, were determined by western blot assays. GAPDH was used as a loading control. (B) Overexpression of FKBPL using Flag tagged FKBPL overexpression plasmid. A549 cells were transiently transfected with either Flag tagged FKBPL overexpression plasmid (FKBPL^OE^) or empty vector control plasmid (Vector), and Flag tagged FKBPL was confirmed by western blot assays. GAPDH was used as a loading control. (C) Ectopic expression of FKBPL decreases the number of colonies in A549 cells. A549 cells were transfected with FKBPL^OE^ or Vector, and colony formation assay was used to demonstrate the inhibitory effect of FKBPL on cell colony formation. (D) Knockdown of FKBPL using control shRNA (shCtrl) or FKBPL shRNAs (shFKBPL#1 and shFKBPL#2). H1975 cells were transiently transfected with either shCtrl plasmid or FKBPL shRNA plasmid, and efficiency of FKBPL knockdown was confirmed by western blot assays. GAPDH was used as a loading control. (E) Knockdown of FKBPL increases the number of colonies in H1975 cells. H1975 cells were transfected with shCtrl or shFKBPL#1, and colony formation assay was used to demonstrate the enhancement effect of FKBPL knockdown on cell colony formation. (F) FKBPL upregulation delays G1/S phase transition, while FKBPL knockdown promotes G1/S phase transition. A549 cells were transfected with FKBPL^OE^ or Vector, H1975 cells were transfected with shCtrl or shFKBPL#1, and cell cycle distribution was determined by flow cytometry. (G) FKBPL upregulation decreased Cyclin E1 expression, while FKBPL silencing enhances Cyclin E1 expression. A549 cells were transfected with FKBPL^OE^ or Vector, H1975 cells were transfected with shCtrl or shFKBPL#1, and protein levels of Cyclin E1 in A549 and H1975 were determined by western blot assays. GAPDH was used as a loading control.

We subsequently explored the effects of FKBPL on the biological behaviors of lung ADC cells. We first performed western blot to examine the expression of FKBPL in two lung ADC cell lines, A549 and H1975. As shown in Fig. 3A, H1975 cells expressed FKBPL at a higher level compared with A549 cells. Previous study demonstrated that FKBPL inhibited proliferation of breast cancer and lymphoma cells 21. We next investigated whether increased FKBPL expression results in inhibition of proliferation of lung ADC cells. We overexpressed FKBPL by ectopically transfecting FKBPL-overexpression (FKBPL^OE^) plasmid into A549 cells. The overexpression of FKBPL was confirmed by western blot analysis (Fig. 3B). Colony formation assay showed that ectopic expression of FKBPL significantly decreased the number of colonies compared with control cells (Fig. 3C). We next tested whether knockdown of FKBPL can promote the proliferation of lung ADC cells. We ablated FKBPL expression by transfecting FKBPL-shRNAs (shFKBPL#1 and shFKBPL#2) into H1975 cells. As shown in Fig. 3D, the two shRNAs were highly effective in depleting FKBPL in H1975 cells. We randomly selected shFKBPL#1 for the subsequent experiments. As anticipated, knockdown of FKBPL markedly increased the number of colonies compared with control cells (Fig. 3E). We further tested the effect of FKBPL on cell cycle distribution. As revealed by Fig. 3F, overexpression of FKBPL increased the proportion of cells in the G1 phase, and decreased the proportion of cells in the S phase in A549 cells. In contrast, inhibition of FKBPL decreased the proportion of cells in the G1 phase, and increased the proportion of cells in the S phase in H1975 cells. We next examined the effect of FKBPL on the protein levels of cell-cycle regulator, Cyclin E1. Western blot analysis showed that overexpression of FKBPL decreased Cyclin E1 expression in A549 cells. In contrast, silencing of FKBPL increased Cyclin E1 expression in H1975 cells (Fig. 3G). Collectively, these data suggest that FKBPL suppresses the proliferation of lung ADC cells by delaying G1/S phase transition.

### 3.4 FKBPL promotes the apoptosis of lung ADC cells

**Figure 4:**
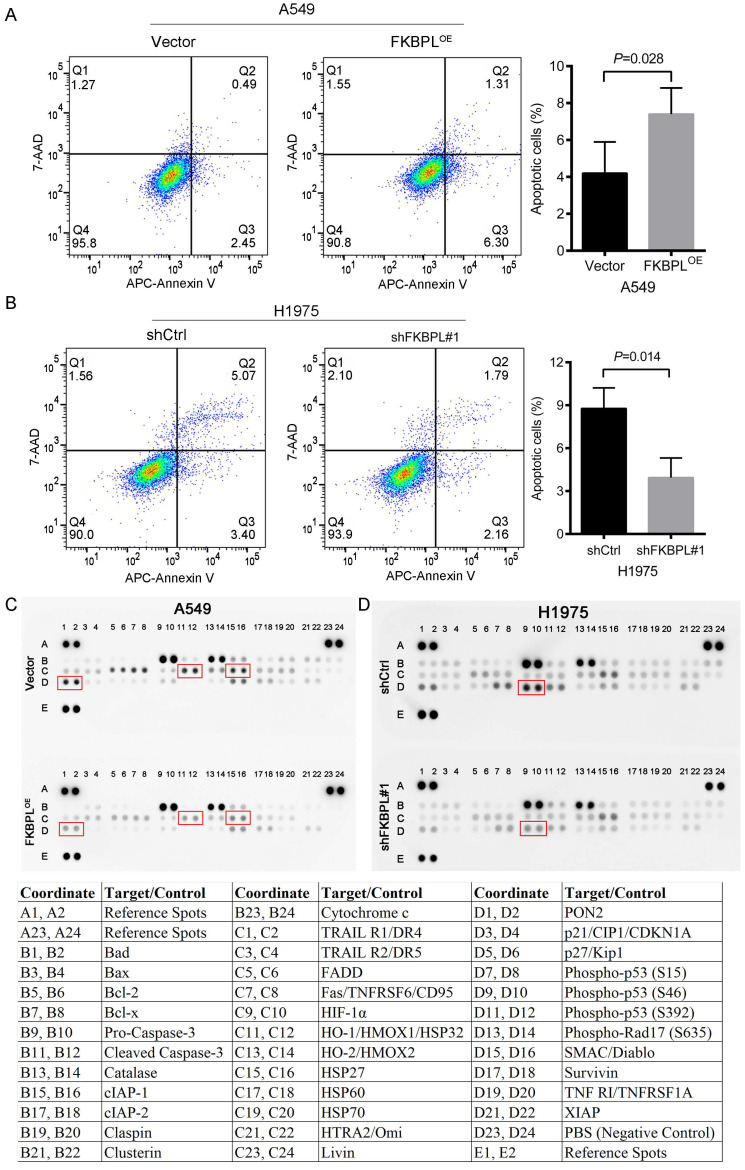
FKBPL promotes the apoptosis of lung ADC cells. (A) FKBPL upregulation promotes the apoptosis of A549 cells. A549 cells were transfected with FKBPL^OE^ or Vector, and cell apoptosis was analyzed by flow cytometry. (B) FKBPL knockdown decreases the apoptosis of H1975 cells. H1975 cells were transfected with shCtrl or shFKBPL#1, and cell apoptosis was analyzed by flow cytometry. (C) Overexpression of FKBPL in A549 cells decreased the anti-apoptotic proteins, including heat shock protein 32 (HSP32), heat shock protein 27 (HSP27), and paraoxonase-2 (PON2). A549 cells were transfected with FKBPL^OE^ or Vector, and the expression levels of apoptosis-related proteins were determined by the proteome profiler human apoptosis array analysis. (D) FKBPL depletion attenuated the pro-apoptotic protein, phospho-p53 (S46) in H1975 cells. H1975 cells were transfected with shCtrl or shFKBPL#1, and the expression levels of apoptosis-related proteins were determined by the proteome profiler human apoptosis array analysis.

We further tested whether FKBPL could regulate the apoptosis of lung ADC cells. As shown in Fig. 4A, ectopic expression of FKBPL resulted in increased apoptosis in A549 cells. In contrast, inhibition of FKBPL led to decreased apoptosis in H1975 cells (Fig. 4B). To further identify proteins contributing to the apoptotic phenotype, the Proteome Profiler Human Apoptosis Array Kit was used to analyze the expression profiles of apoptosis-related proteins. We observed that overexpression of FKBPL in A549 cells significantly decreased the anti-apoptotic proteins, including heat shock protein 32 (HSP32), heat shock protein 27 (HSP27), and paraoxonase-2 (PON2) (Fig. 4C). FKBPL depletion significantly attenuated the pro-apoptotic protein, phospho-p53 (S46) in H1975 cells (Fig. 4D). Together, our data indicate that FKBPL promotes the apoptosis of lung ADC cells.

## 4. Discussion

FKBPL, also known as WAF-1/CIP1 stabilizing protein 39, is a divergent member of the FK506-binding protein (FKBP) family. It shares homology with the FKBP family mostly in the C-terminal tetra-trico-peptide repeat (TPR) domain, but lacks the crucial residues within a weakly homologous peptidyl prolyl cis/trans isomerase (PPIase) domain that are required for enzymatic activity 21.

FKBPL has been demonstrated to regulate steroid receptor and inflammatory signalling via CD44, heat shock protein 90 (HSP90) and signal transducer and activator of transcription-3 (STAT-3) 10. Upon binding to HSP90, FKBPL regulates the stability of p21 and forms co-chaperone complexes with GR, AR and ERα 22. Heat shock proteins are a large family of evolutionarily preserved molecular chaperones, and key regulators of post-translational protein folding. Dysregulated expression of heat shock protein has been demonstrated to play crucial roles in tumour development. Heat shock proteins are reported to be involved in hallmarks of cancer, such as mitosis, and apoptosis 23. Aberrant expression of HSP27 is associated with tumorigenesis and metastasis in various cancers. It has been identified as a regulator of the Hippo signalling pathway, which controls cancer initiation/progression, organ development, and stem cell maintenance and regeneration 24, 25. HSP27 prevents apoptosis by associating with caspase-3, cytochrome c, Bax, IκB kinase (IKK), and others 26. HSP32, also known as heme oxygenase 1 (HO-1), is closely related to participation in diminishing oxidative stress damage and exerting anti-apoptotic effects 27. In this study, we demonstrate that, overexpression of FKBPL in A549 cells significantly decreased the anti-apoptotic proteins, including HSP32, and HSP27. The tumour suppressor p53 regulates various pathways, including those involved in DNA damage, and cytostatic/cytotoxic responses. P53 exerts pro-apoptotic activity in transcription-dependent and independent manners. As a transcription factor, p53 directly regulates the transcription of hundreds of genes, leading to cell-cycle arrest and apoptosis. P53 also enhances apoptosis in a transcription independent manner by activating the mitochondria death pathway 28, 29. It has been reported that p53 phosphorylation at Ser46 is critical for apoptosis induction. Mdm2-dependent homeodomain-interacting protein kinase 2 (HIPK2) degradation prevents phosphorylation of p53 at Ser46, which ultimately leads to cell survival 30. In the present study, FKBPL depletion significantly attenuated phospho-p53 (S46) in H1975 cells. It was reported that FKBPL overexpression decreases proliferation and clonogenicity of breast cancer cells via stabilizing newly synthesized p21. Moreover, FKBPL overexpression inhibits proliferation of leukemic U937 cells by delaying cells in the G0-G1 phase of the cell cycle 14. Our study suggests that FKBPL can suppress the proliferation of lung ADC cells by delaying G1/S phase transition.

FKBPL has been shown to function as a tumour suppressor in various types of human malignancies. In the present study, we showed that FKBPL was significantly lower in lung ADC than the normal tissues. Patients with well or moderately differentiated tumours have higher FKBPL expression compared with patients with poor differentiated tumours. However, no significant associations were found between FKBPL expression and other clinicopathological variables. Previous studies demonstrated that high FKBPL expression was significantly correlated with prolonged OS and distant metastasis-free survival in patients with breast cancer 13, 14. Moreover, high endogenous tumour expression of FKBPL was significantly associated with increased progression-free survival in patients with high-grade serous ovarian cancer 16. Our immunohistochemistry study showed that high FKBPL expression was correlated with prolonged OS in patients with lung ADC. However, no significant associations were found between FKBPL expression and OS according to the Human Protein Atlas and Kaplan-Meier plotter databases. The Human Protein Atlas is an online database containing large sets of protein expression data from immunohistochemically stained TMAs 31. The Kaplan-Meier plotter is an online survival analysis database for 54,000 genes in 21 malignant tumours 32. The observed discrepancy between our immunohistochemistry study and the Human Protein Atlas and Kaplan-Meier plotter database analysis could be explained by the fact that our immunohistochemistry data are from a single center, which might have led to inherent biases and requires further external validation.

Taken together, our study revealed that high FKBPL expression was correlated with prolonged OS in patients with lung ADC. Moreover, FKBPL could suppress the proliferation of lung ADC cells by delaying G1/S phase transition. Our study also demonstrated that FKBPL overexpression could promote the apoptosis of lung ADC cells. These new findings provide an experimental basis for further theoretical investigation of lung ADC.

## Supplementary Material

Supplementary figures.

## Figures and Tables

**Table 1:** Relationship between expression levels of FKBPL and clinicopathological features of 222 lung ADC specimens

Variables	n	FKBPL		*P* value
		Low	High	
Age (years)				0.779
<60	65	39	26	
≥60	157	91	66	
Gender				0.703
Female	124	74	50	
Male	98	56	42	
Smoking status				0.310
Never smoked	171	97	74	
Ex-smoking or currently smoking	51	33	18	
Differentiation				0.037
Well/moderate differentiation	139	74	65	
Poor differentiation	83	56	27	
T stage				0.653
T1/T2	208	121	87	
T3/T4	14	9	5	
N stage				0.174
N0	158	88	70	
N1/N2/N3	64	42	22	
TNM stage				0.125
I/II	180	101	79	
III	42	29	13	
Lymphovascular invasion				0.378
Negative	152	86	66	
Positive	70	44	26	
Tumor spread through air spaces (STAS)				0.441
Negative	109	61	48	
Positive	113	69	44	

A *P* value < 0.05 was considered significant.

**Table 2:** Univariate and multivariate analysis of prognostic factors in 222 lung ADC specimens

	Univariate analysis			Multivariate analysis	
	HR (95% CI)	*P*		HR (95% CI)	*P*
Age (≥60 *vs.* <60)	0.925 (0.537 to 1.595)	0.780		-	-
Gender (male *vs.* female)	0.610 (0.367 to 1.015)	0.057		-	-
Smoking status (ex-smoking or currently smoking *vs.* never smoked)	1.156 (0.644 to 2.074)	0.628		-	-
Differentiation (poor differentiation *vs.* well/moderate differentiation)	2.477 (1.488 to 4.124)	<0.001		1.295 (0.703 to 2.384)	0.407
T stage (T3/T4 *vs.* T1/T2)	1.715 (1.181 to 2.489)	0.005		1.562 (1.054 to 2.315)	0.026
N stage (N1/N2/N3 *vs.* N0)	5.040 (2.997 to 8.475)	<0.001		4.397 (2.465 to 7.843)	<0.001
Lymphovascular invasion (positive *vs.* negative)	1.709 (1.008 to 2.895)	0.047		0.889 (0.495 to 1.594)	0.692
STAS (positive *vs.* negative)	1.985 (1.167 to 3.375)	0.011		1.099 (0.592 to 2.040)	0.764
FKBPL (high *vs.* low)	0.471 (0.265 to 0.835)	0.010		0.575 (0.321 to 1.030)	0.063
