# Supplementary figures and images for: Expression and role of FKBPL in lung adenocarcinoma
Source: J Cancer. 2024 Jan 1;15(1):166–75. doi: 10.7150/jca.87758 (PMC10751668; doi:10.7150/jca.87758)

Supplementary Figure 1

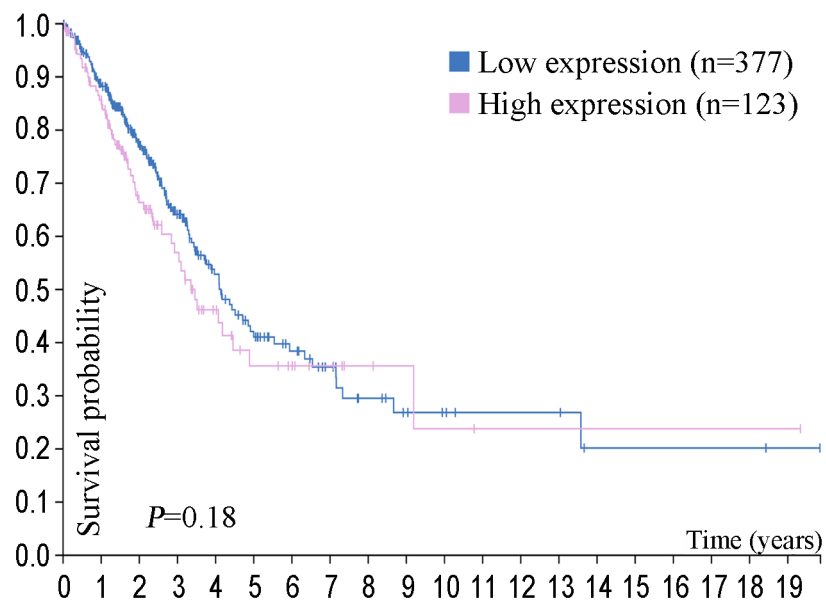

Supplementary Figure 2

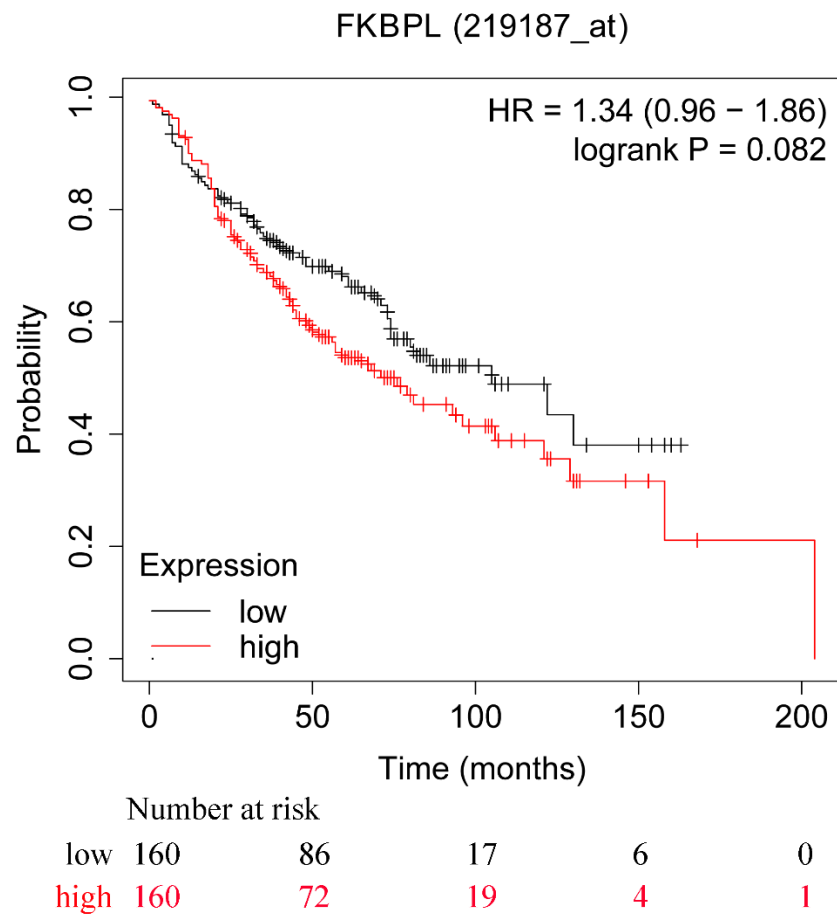

Supplement: Supplementary file 1 — Supplementary figures. [file jcav15p0166s1.pdf]
